# Anti-Inflammatory and Analgesic Activities of a Novel Biflavonoid from Shells of *Camellia oleifera*

**DOI:** 10.3390/ijms131012401

**Published:** 2012-09-27

**Authors:** Yong Ye, Ya Guo, Yue-Ting Luo

**Affiliations:** Pharmaceutical Engineering Department, School of Chemistry and Chemical Engineering, South China University of Technology, Guangzhou 510640, China; E-Mails: guoyaqz@163.com (Y.G.); skating009@yahoo.cn (Y.-T.L.)

**Keywords:** biflavonoid, *Camellia oleifera*, anti-inflammation, analgesic activity, antioxidative activity

## Abstract

Shells are by-products of oil production from *Camellia oleifera* which have not been harnessed effectively. The purpose of this research is to isolate flavonoid from shells of *Camellia oleifera* and evaluate its anti-inflammatory and analgesic effects. The flavonoid was identified as bimolecular kaempferol structure by UV, MS, ^1^H NMR and ^13^C NMR spectra, which is a new biflavonoid and first found in *Camellia oleifera*. It showed dose-dependent anti-inflammatory activity by carrageenin-induced paw oedema in rats and croton oil induced ear inflammation in mice, and analgesic activity by hot plate test and acetic acid induced writhing. The mechanism of anti-inflammation of biflavonoid is related to both bradykinin and prostaglandins synthesis inhibition. The biflavonoid showed both central and peripheral analgesic effects different from aspirin, inhibition of the synthesis or action of prostaglandins may contribute to analgesic effect of biflavonoid. The biflavonoid significantly decreased malonaldehyde (MDA) and increased superoxidase dismutase (SOD) and Glutathione peroxidase (GSH-Px) activity in serum (*p* < 0.01), revealed strong free radical scavenging activity *in vivo*. It indicates the biflavonoid can control inflammation and pain by eliminating free radical so as to inhibit the mediators and decrease the prostaglandins. The biflavonoid can be used as a prospective medicine for inflammation and pain.

## 1. Introduction

Pain is a kind of symptom and disease with emotional changes and tissue damage. Instant relief is essential since severe pain can cause metabolism disorder and other diseases [1]. Analgesics are mainly divided into two classes: opiate receptor agoists and non-steroidal anti-inflammatory drugs (NSAIDs). The former may lead to drug dependence even though it is effective for various pains. NSAIDs are widely used in the treatment of pain, but they have side effects, especially on the gastro intestinal tract [2]. New analgesics with little side effects have become the focus of recent research.

Inflammation as one major cause of pain is related to cancer, diabetes and cardiovascular disease [3]. It is the body’s immediate response to damage its tissues and cells by pathogens, noxious substances, or physical injury [4]. These instigators induce activation of inflammatory mediators such as kinins, cyclooxygenase products and cytokines, which have become key targets for therapeutic intervention in a range of diseases including pain [5].

Flavonoids are polyphenols widely distributed in plants. There are about 5000 kinds of flavonoids, including flavones, biflavonoids, flavanones, flavanonols, isoflavones, flavan-3-ols, chalcones, flavan-3,4-diols, dihydrochalcones, anthocyanidins, xanhones, *etc.*, which have the function of protecting cardiovascular, antioxidant, anti-tumor, relieving cough and phlegm, anti-inflammation, anti-virus and adjusting immunity, *etc.* [6–8]. Flavonoids were also found in *Camellia oleifera*, which have antioxidative activity as well [9,10], but have not been manufactured; their pharmacological activities are less reported.

The shells of *Camellia oleifera* are by-products of oil production, which are always discarded or used as fertilizer. Many bioactive compounds such as saponins, flavonoid glycosides and polysaccharides are found in the seeds [11–15], anti-inflammatory and analgesic effect of saponins are also disclosed [16], but little is known about the shells. Preliminary studies have also revealed the free radical scavenging activity of the total flavonoids from shells [17], however, the flavonoid is a mixture at low purity. In order to purify the flavonoid and analyze its pharmacological activity, we use a simple method to isolate the flavonoid from the shells, a novel biflavonoid was found and its anti-inflammatory and analgesic activities were further elucidated.

## 2. Results and Discussion

### 2.1. Separation and Identification of Flavonoid

The total flavonoid content was about 2.3% on basis of standard curve of rutin. The linear regression equation was *y* (rutin, mg/mL) = 0.015*A* (absorbance) + 0.0013 (*r* = 0.9996). The flavonoid of *Camellia oleifera* was extracted by methanol, hydrolyzed and precipitated by hydrochloric acid. High purity of flavonoid could be obtained when the deposit was crystallized by aqueous acetone. Purity of the flavonoid was up to 93.8% which was calculated on peak area by HPLC, and yield was 2.1%. This means the extraction ration of flavonoid is 85.6%. General precipitation and extraction could purify the flavonoid from shells of *Camellia oleifera* without column chromatography, which is proper for industrial production.

**Figure 1:**
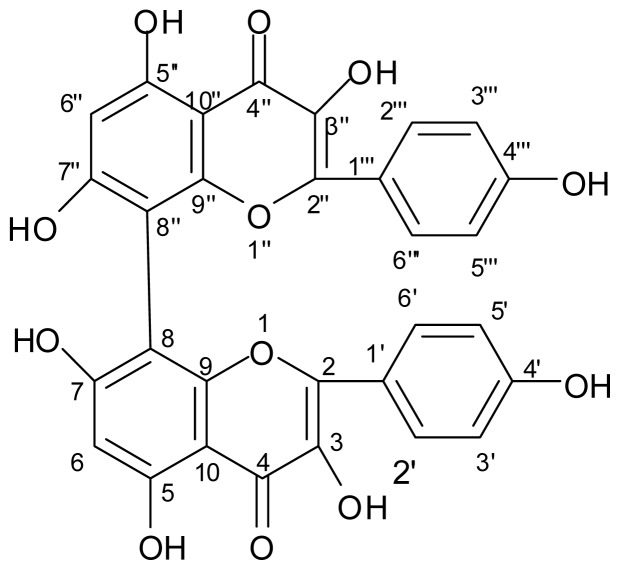
Molecular structure of the biflavonoid extracted from shells of *Camellia oleifera*.

The IR spectra showed the characteristic absorption band of hydroxyl (3423 cm^−1^), aromatic ring (1514, 1459 cm^−1^) and conjugated carbonyl (1648 cm^−1^). There were 3 absorption peaks of UV (207, 265, 366 nm) due to chromophore of flavonoid. *m*/*z* [M]^+^ : 570.9, molecular formula could be deduced to C_30_H_18_O_12_. In ^1^H NMR (400 MHz, DMSO-d_6_) spectra, δ12.48 (s, br, 2H), 10.78 (s, br, 2H), 10.11 (s, br, 2H), 9.37 (s, br, 2H) were 4 signals of hydroxyl protons on aromatic rings of kaempferol, δ8.04 (d, *J* = 8.8 Hz, 4H) and 6.93 (d, *J* = 8.8 Hz, 4H) formed two AA′BB′ systems, δ6.44 (s, 2H) was 2 protons on benzene rings of kaempferol. ^13^C NMR (101 MHz, DMSO-d_6_) spectra showed 2 carbonyl carbons, 14 double bonds with 28 carbons. The shift of C-8(8″) with δ4.2 compared to kaempferol suggested direct C–C bond of C-8 and C-8″ [18]. The correlation of C and H was further confirmed by HMBC spectra (Table 1). Thus the compound could be deduced to bimolecular kaempferol structural biflavonoid (Figure 1).

There are many flavonoid glycosides in the seeds of *Camellia oleifera*, whose basic part is kaempferol, but bimolecular kaempferol compound has not been found so far. Kaempferol glycosides can be obtained through separation [19], but pure kaempferol glycoside could not be easily gained unless column chromatography is applied. By ways of solvent extraction, hydrolysis, precipitation and crystallization, high purity of biflavonoid with bimolecular kaempferol structure was acquired in our research. The method is suitable for isolation of biflavonoid from the shells of *Camellia oleifera* in industry.

### 2.2. Effect of the Biflavonoid on Carrageenin Induced Oedema in Rats

The biflavonoid from shells of *Camellia oleifera* gave significant (*p* < 0.01) reduction of carrageenin-induced paw oedema at the interval of 2 h and 4 h, but no difference at 1 h as compared to saline control (Table 2). The effect increased in a dose dependent manner. The maximum inhibition of inflammation was up to 60.3% at a dose of 200 mg/kg (i.g.), whereas the aspirin showed 58.1% of inhibition at the same dose.

### 2.3. Inhibition on Croton-Oil Induced Ear Inflammation in Mice

The similar result was obtained from croton-oil induced ear inflammation in mice, the biflavonoid significantly (*p* < 0.01) decreased the weight of inflamed ear (Table 3). At the same dose of 200 mg/kg (i.g.), the biflavonoid inhibited inflammation up to 71.9%, slightly higher than that of aspirin. The effect depended on the dosage.

The biflavonoid has made obvious anti-inflammatory effects in Carrageenin induced oedema in rats and croton-oil induced ear inflammation in mice. The two methods are widely used in screening of anti-inflammatory agents [20]. The process of carrageenin-induced inflammatory in the rat involves three phase by several mediators released in sequence [21]. The initial phase during the first 1.5 h is attributed to the release of histamine and serotonin, the second phase is mediated by bradykinin from 1.5 to 2.5 h and the third phase is due to the release of prostaglandins from 2.5 to 6 h after carrageenin injection [22]. The biflavonoid took effects in the second and third phase, it suggested the mechanism of anti-inflammation of biflavonoid is related to both bradykinin and prostaglandins synthesis inhibition.

### 2.4. The Variation of Latency Responses in Hot Plate Test of Mice

**Figure 2:**
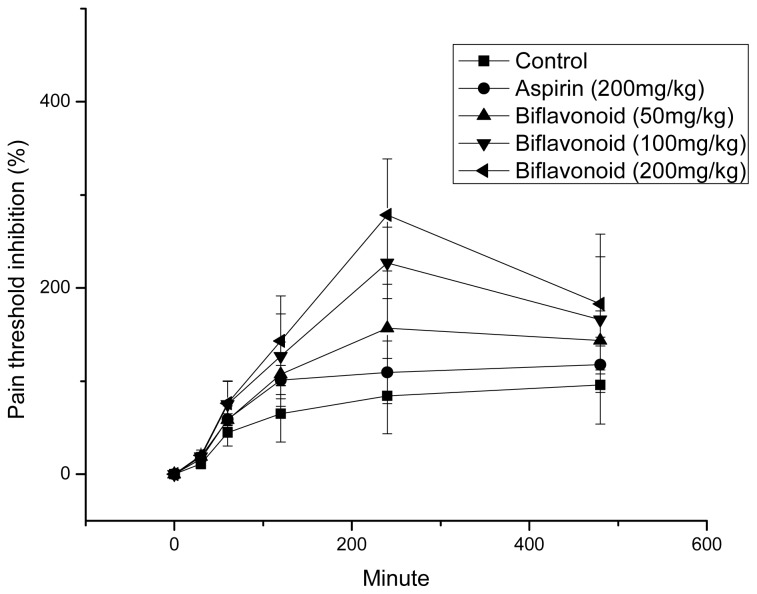
Inhibition of pain threshold in hot plate test of mice by different treatment. Values represent means ± SD (*n* = 8).

Mice pretreated with biflavonoid showed a dose dependent increase in latency of response in the hot plate method (Figure 2). The latency responses increase with time and reached maximum at time interval of 4 h, when the pain threshold inhibition were significant (*p* < 0.01) at dose of 100 and 200 mg/kg of biflavonoid as compared to control, and the effect increased remarkably (*p* < 0.01) compared with aspirin.

### 2.5. Control on Acetic Acid-Induced Writhing Responses in Mice

Administration of biflavonoid decreased the number of writhing in mice and the effect was found to be dose dependent. The reduction was significant (*p* < 0.01) when compared to control. The maximum inhibition of writhing was 54% at dose of 200 mg/kg of biflavonoid, the effect was slightly lower than aspirin. The results are given in Table 4.

Hot plate test and acetic acid-induced writhing are commonly used in evaluation of analgesic effect. The former is thermal induced nociception indicating narcotic involvement [23], which is undertaken to verify if agents have any central analgesic effect. The latter is chemical induced nociception for detecting both central and peripheral analgesics. Intraperitoneal injection of acetic acid leads to high levels of prostaglandins in peritoneal exudates [24]. The abdominal constrictions produced after administration of acetic acid is related to sensitization of nociceptors to prostaglandins. Aspirin showed analgesic effect only in writhing test but the biflavonoid showed an effect in both of the two models, it is therefore possible that biflavonoid has both central and peripheral analgesic effects different from aspirin. Inhibition of the synthesis or action of prostaglandins may contribute to analgesic effect of biflavonoid.

### 2.6. Effect of Biflavonoid on MDA, SOD and GSH-Px Activity

MDA, SOD and GSH-Px are always used to deduce free radical levels in vivo [25]. A significant (*p* < 0.01) reduction of MDA level and increase of SOD and GSH-Px activities in rat blood were observed with biflavonoid at dose of 100 and 200 mg/kg compared with control (Table 5). The biflavonoid caused greater changes than aspirin at the same dose, and it revealed biflavonoid from *Camellia oleifera* was more effective in eliminating free radical in vivo than aspirin.

Many researches prove the correlation between inflammation and free radicals which is regarded as the main cause of damage from inflammation [26]. Free radicals with one or more unpaired outer shell electrons are extremely reactive and generally highly unstable. Reactive oxygen species, such as superoxide radical, hydrogen peroxide, hydroxyl radical, and singlet oxygen are main free radicals in body and of the greatest biological significance. They are extremely reactive and potentially damaging transient chemical species. Free radicals generated by mitochondria metabolism can be removed by redox system in vivo in healthful condition, however, the balance is broken in illness, and excess free radicals keep active, injury normal tissues and activate inflammatory mediators. Free radicals *in vivo* can be evaluated by MDA, SOD and GSH-Px activities. MDA is lipid peroxide produced by cells at the imbalance of oxidative and antioxidative system can combine phospholipin, protein and nucleic acid to form stable and insoluble substance which can induce cell damage. SOD and GSH-Px which can protect integrity of cell membrane structure and function, and eliminate free radicals, are important antioxidative enzymes. In this experiment, biflavonoid from the defatted shells of *Camellia oleifera* showed strong capacity of eliminating free radicals in blood by promoting activities of SOD and GSH-Px, but aspirin did not. The elimination of free radicals leads to reduction of inflammatory mediators and inhibition of prostaglandins. Therefore, free radical scavenging ability of the biflavonoid may be responsible for its anti-inflammatory and analgesic property. Even though aspirin has anti-inflammatory and analgesic activity but its mechanism is different.

The biflavonoid from the defatted shells of *Camellia oleifera* has two molecular kaempferol, will perform stronger pharmacological effects than one molecular kaempferol which is found in many plants and has broad pharmacological activity as reported [27]. It is a good candidate for anti-inflammatory medicine and analgesics. Other functions of the bi-kaempferol need further investigation.

## 3. Experimental Section

### 3.1. Plant Material

The shells of *Camellia oleifera* were collected from Guangdong Tea Oil Company in Meizhou of Guangdong province, China.

### 3.2. Chemicals and Reagents

Superoxidase dismutase (SOD) kit, Malondialdehyde (MDA) kit, and Glutathione peroxidase (GSH-Px) kit were purchased from Nanjing Jiancheng Bioengineering Institute (Nanjing, China); Aspirin was produced by Wuhan Chemical Co. Ltd; Rutin was bought from Shanghai Chemical Co. Ltd; Carrageenin, acetic acid and other chemical reagents were purchased from Qianhui Chemical Company (Guangzhou, China).

### 3.3. Animals

The experiments were carried out on Wistar rats weighing 180–200 g and Kunming mice (male or female) weighing 18–22 g, The animals were housed under conditions of 25 ± 2 °C, 50% ± 10% humidity with a 12 h light/dark cycle. All animals used in this work were treated according to the guideline of animal handling in South China University of Technology.

The animals were divided into 5 groups of 6–8 animals each in all tests. Group 1 served as control and received normal saline (1 mL/100 g), group 2 received Aspirin (200 mg/kg) as positive. Group 3–5 respectively received biflavonoid at dose of 50, 100, 200 mg/kg. Aspirin and biflavonoid were suspended in normal saline and intragastricly administered.

### 3.4. Extraction and Isolation

The shells of *Camellia oleifera* (1 kg) was refluxed in 10 L of 70% methanol at 80 °C for 2 h. The filtrate was vacuum concentrated and dried at 60 °C, 150 g of solid extract was hydrolyzed in 2.4 L of hydrochloric acid (2.0 M) under reflux at 80 °C for 5 h. The precipitation was centrifuged in 3000 rpm for 10 min, and washed with 500 mL of water for 3 times, then crystallized in 150 mL of 80% acetone aqueous solution. The crystal was air-dried in hood at room temperature; 21 g of yellow powder was obtained.

### 3.5. Determination of Total Flavonoid Content

Total flavonoid content was determined according to Zhishen *et al.* [28]. 0.1 g of the dried shell powder was extracted with 250 mL of 75% methanol under reflux for 1 h. 10 mL of the filtrate was mixed with 1 mL of 10% aluminum chloride and 5% sodium nitrite, and a pink-colored flavonoid-aluminum complex was formed after 10 mL of 1 M sodium hydrate was added to the solution. The absorbance at 510 nm was determined 5 min after the mixing of the solution. A reagent blank containing methanol instead of sample was used. The total flavonoid content was calculated using a standard curve of rutin.

### 3.6. Determination of Purity by HPLC

The analysis was run on HP 1100 HPLC (Agilent Company, Santa Clara, CA, USA). The operating condition was column, Hypersil ODS (250 × 4.6 mm, 5 μm); flow phase, methanol *vs.* water (60:40); injection volume, 20 μL; flow rate, 1 mL/min; temperature, 30.0 °C; wavelength, 366 nm.

### 3.7. Structure Identification

UV spectra analysis was carried out on UV-3010 Ultra violet spectrometer (Hitachi company, Tokyo, Japan) scanning from 200 to 600 nm; IR spectra were measured on Nicolet 380 FI-IR spectrograph (Nicolet apparatus company, Madison, WI, USA) with KBr tablets from 4000 to 400 cm^−1^ with resolution 2 cm^−1^; Mass spectra were recorded on Bruker Esquire Hct Plus Mass spectrometer with ESI (Bruker company, Bremen, Germany) in m/z of cation model scanning from 150 to 1200 for 60 min; NMR spectra were determined on 400 MHz AM NMR (Bruker company, Faelladen, Switzerland) in DMSO-d_6_ operating at 101 MHz for ^13^C NMR and 400 MHz for ^1^H NMR.

### 3.8. Anti-Inflammatory and Antioxidative Test

Oedema was produced by the method as described [29]. Aspirin and flavonoid extract were administered 30 min prior to injection of 0.1 mL/100 g carrageenin (10 mg/mL) into the plantar aponeurosis of right hind paw of the rats of all groups. The paw volume was measured initially and at 1, 2, 4 h using a plethysmometer after carrageenin injection. Blood was taken from tail cut, and serum was separated and used to determine MDA, SOD and GSH-Px according to the kit description.

### 3.9. Croton-Oil Induced Ear Inflammation

Croton oil irritant solution (0.2 mL) was prepared according to the reference [30] and applied to the innersurface of the right ear of mice. Aspirin and flavonoid extract were administered 30 min before croton oil application. 4 h later, the mice were sacrificed by cervical dislocation and 7 mm punches were made in the ear by a cork borer. Each ear disc was weighed; the difference between right and left punched ear was calculated as swelling value. Anti-inflammatory activity was recorded as percent inhibition of swelling value in control.

### 3.10. Hot Plate Test

The analgesic effect was assessed by the method described by Somchit *et al.* [31]. The latency time for paw licking was recorded as pain threshold when mice were exposed to the hot plate surface which is kept at 55 ± 1 °C. Basic pain threshold was measured, then all treatments were given 30 min before the thermal stimulus and the response was determined at 30, 60, 120, 240 and 480 min. Pain threshold inhibition (%) = (*P*_t_ − *P*_0_) × 100/*P*_0_, *P*_0_ and *P*_t_ separately presents basic pain threshold and pain threshold at time interval.

### 3.11. Acetic Acid-Induced Writhing

This test was performed as described by Fontenele *et al.* [32]. The writhes were induced by intraperitoneal injection of acetic acid (0.6%, *v*/*v*) at 0.1 mL/10 g. Aspirin and flavonoid extract were administered 30 min before acetic acid injection. The number of abdominal contraction was counted over a period of 20 min and expressed as writhing numbers. Antinociceptive activity was expressed as percent inhibition of writhing number in control animals.

### 3.12. Statistical Analysis

Data was showed as mean ± standard derivation (SD). SPSS 11.0 software (SPSS Inc., Chicago, IL, USA, 1992) was used to analyze the data of animal tests in groups by AVOVA and Dunnett’s tests.

## 4. Conclusions

The biflavonoid with bimolecular kaempferol structure has been found in the shells of *Camellia oleifera*. The separation procedure includes reflux extraction, hydrolysis and crystallization, which is simple and proper for industrial production. The biflavonoid has obvious anti-inflammatory effect. It can control pain induced by heat and chemicals indicating its central and peripheral analgesic effects. The anti-inflammatory and analgesic mechanism may be attributed to inhibition of the synthesis or action of prostaglandins, which is related to its ability of eliminating free radical *in vivo*.

## Figures and Tables

**Table 1:** ^1^H NMR, ^13^C NMR and HMBC data of the biflavonoid from shells of *Camellia oleifera* (DMSO-d_6_).

Position	^1^H NMR	^13^C NMR	HMBC
2(2″)		146.8	
3(3″)		135.6	
4(4″)		176.9	
5(5″)		160.7	
6(6″)	6.44 (s)	98.2	C-5(5″),7(7″),8(8″),10(10″)
7(7″)		163.9	
8(8″)		98.2	
9(9″)		156.2	
10(10″)		103.0	
1′ (1‴)		121.6	
2′(2‴)	8.04 (d, *J* = 8.8 Hz)	129.5	C-1′(1‴),3′(3‴),5′(5‴)
3′(3‴)	6.93 (d, *J* = 8.8 Hz)	115.4	C-2′(2‴),4′(4‴),6′(6‴)
4′(4‴)		159.2	
5′(5‴)	6.93 (d, *J* = 8.8 Hz)	115.4	C-2′(2‴),4′(4‴),6′(6‴)
6′(6‴)	8.04 (d, *J* = 8.8 Hz)	129.5	C-1′(1‴),3′(3‴),5′(5‴)
3-OH(3″-OH)	10.78 (s, br)		C-2(2″),3(3″),4(4″)
5-OH(5″-OH)	12.48 (s, br)		C-5(5″),6(6″),10(10″)
7-OH(7″-OH)	10.10 (s, br)		C-6(6″),7(7″),8(8″)
4′-OH(4‴-OH)	9.37 (s, br)		C-3′(3‴),4′(4‴),5′(5‴)

**Table 2:** Effect of the biflavonoid on carrageenin-induced paw oedema in rats.

Drug	Dose (mg/kg, i.g.)	Difference in volume between right paw and left paw in mL Mean ± SD (*n* = 6)			

		0 h	1 h	2 h	4 h
Normal control	/	0.14 ± 0.03	0.19 ± 0.03	0.29 ± 0.04	0.53 ± 0.05
Aspirin	200	0.13 ± 0.02	0.17 ± 0.03	0.21 ± 0.03 **	0.22 ± 0.03 **
Biflavonoid	50	0.15 ± 0.04	0.18 ± 0.04	0.24 ± 0.04	0.36 ± 0.05
Biflavonoid	100	0.15 ± 0.04	0.17 ± 0.04	0.24 ± 0.04	0.28 ± 0.05 **
Biflavonoid	200	0.12 ± 0.02	0.16 ± 0.02	0.21 ± 0.03 **	0.21 ± 0.02 **

Data were analyzed by ANOVA and Dunnett’s test.

** Denotes significant inhibition as compared to normal control (*p* < 0.01).

**Table 3:** Effect of the biflavonoid on croton oil induced ear inflammation in mice.

Drug	Dose (mg/kg, i.g.)	Difference in weight between left and right punched ear in mg Mean ± SD (*n* = 8)	Inhibition (%)
Normal control	/	36.1 ± 9.3	/
Aspirin	200	11.0 ± 5.0 **	69.5
Biflavonoid	50	24.5 ± 4.6	32.2
Biflavonoid	100	18.6 ± 4.4 **	48.6
Biflavonoid	200	10.2 ± 3.6 **	71.9

Data were analyzed by ANOVA and Dunnett’s test.

** Denotes significant inhibition as compared to normal control (*p* < 0.01).

**Table 4:** Inhibition on acetic acid induced writhing in mice by the biflavonoid.

Drug	Dose (mg/kg, i.g.)	Number of writhing Mean ± SD (*n* = 8)	Inhibition (%)
Normal control	/	72.6 ± 7.6	/
Aspirin	200	22.6 ± 4.6 **	68.8
Biflavonoid	50	57.2 ± 9.0	21.2
Biflavonoid	100	48.5 ± 6.5 **	33.2
Biflavonoid	200	33.4 ± 5.7 **	54.0

Data were analyzed by ANOVA and Dunnett’s test.

** Denotes significant inhibition as compared to normal control (*p* < 0.01).

**Table 5:** Effect of the biflavonoid on malonaldehyde (MDA), superoxidase dismutase (SOD) and Glutathione peroxidase (GSH-Px) activities in blood of rats.

Groups	Dose (mg/kg)	MDA (nmol/mL)	SOD (U/mL)	GSH-Px (U/mL)
Normal control		6.11 ± 0.47	61.62 ± 7.92	41.32 ± 8.54
Aspirin	200	5.42 ± 0.77	60.59 ± 5.27	48.08 ± 7.33
Biflavonoid	50	5.47 ± 0.96	59.20 ± 7.66	52.53 ± 8.12
Biflavonoid	100	4.70 ± 0.88 **	67.36 ± 6.32	56.62 ± 8.76 **
Biflavonoid	200	3.70 ± 0.92 **	75.43 ± 5.95 **	78.12 ± 8.79 **

Each value was expressed as mean ± standard deviation (*n* = 6), data were analyzed by ANOVA and Dunnett test.

** Significant difference at *p* < 0.01, compared with normal control.
